# Endodontic radiography - what’s displaying the radiograph? The yield of commercial computer screens vs. DICOM calibrated medical screens in endodontic radiography

**DOI:** 10.1038/s41405-024-00247-y

**Published:** 2024-07-27

**Authors:** Idan Stiklaru, Ella Lalum, Avi Levin, Avi Shemesh, Hadas Azizi, Nirit Yavnai, Joe Ben Itzhak, Michael Solomonov

**Affiliations:** 1 Department of Endodontics, Israel Defense Forces (IDF), Medical Corps, Tel Hashomer, Israel; 2 “Bina” Program, Faculty of Dental Medicine, Hebrew University of Jerusalem, Jerusalem, Israel; 3 Department of Community Dentistry, Faculty of Dental Medicine, Hebrew University, Hadassah Ein Kerem Campus, Jerusalem, Israel

**Keywords:** Digital radiography in dentistry, Root canal treatment

## Abstract

**Objective:**

A comparison between commercial computer screens and DICOM-calibrated medical screens for characterizing anatomy and diagnosing dental pathologies was performed. The aim of this study was to evaluate and compare the ability of each of those screens to identify root apices and widening of the periodontal ligament (PDL) in the posterior maxillary area.

**Materials and Methods:**

Digital X-ray images of 53 maxillary molar teeth were examined by means of a commercial computer screen and again two months later with a DICOM screen to compare their ability to help identify and diagnose PDL widening and to locate the root apices of those teeth.

**Results:**

The DICOM screen had a significantly better ability to identify widened PDLs (from 31.4% to 34.8% of the cases compared to 19% to 26.1% for the commercial screens, *P* < .001), depending upon the observer. The DICOM screen was also significantly superior in depicting the root apices compared to the commercial screens (from 77.4% to 83.6% of the cases compared to 56% to 66.7% for the commercial screens, *P* < 0.001), depending upon the observer.

**Conclusion:**

DICOM-calibrated medical screens were significantly superior to commercial computer screens for identifying widened PDLs and locating the root apex in the posterior maxillary area.

## Introduction

Digital radiography has become an essential part of dentistry in recent decades. Imaging monitoring is a key component of digital radiology. The monitor should be able to display the maximal number of authentic details of an X-ray image, given that failure may result in missed opportunities for intervention and, on other occasions, perhaps overtreatment [1]. Many studies in the field of medical radiology have investigated the effects of the monitor’s diagnostic capabilities, such as in mammography and chest radiography [2–5]. The results of those studies led the American College of Radiologists and the National Electrical Manufacturers Association to set standards for the display functions. For example, the DICOM standard (DS) defines how grayscale standard display images must be shown in a manner by which they can be displayed consistently [6]. In the early days of digital radiology, commercial screens (CSs) lacked the resolution and brightness needed for radiological image display. Low-quality displays may result in misdiagnosis, as well as fatigue and eyestrain on the part of the radiologists. This led to the development of the new industry of medical-grade computer screens [7], and many medical imaging workstations now employ DS-compatible display monitors as their main diagnostic screen. Several studies have demonstrated that DS improves practitioners’ ability to identify carious lesions and other dental pathologies [1, 8]. To the best of the authors’ knowledge, this subject has not been investigated in the field of endodontology. Ørstavik observed that the posterior maxillary region is a complex area for interpretation by two-dimensional X-ray due to the superimposition of anatomical structures (e.g., the maxillary sinus) [9, 10]. The aim of the current study was to compare the efficacy and accuracy of DS with those of CS in the identification of anatomical structures and dental pathologies as depicted on posterior maxillary periapical digital dental X-rays. The null hypothesis was that the identification abilities of both computer screens would be similar.

## Materials and Methods

### Observer calibration

Randomly selected digital X-ray images of 30 maxillary molar teeth (90 roots) from the authors’ institutional dental records for 2020 were examined by 3 endodontists (authors HA, AL, and AS) and one postgraduate student (author IS) to measure the distance (in mm) from the tip of an instrument (in length X-rays) or from the most apical point of the obturation material (in periapical images of treated teeth) to the anatomical apex of the target tooth and thus serve as a calibration process. Distance was measured using the built-in “ruler” tool in the Synapse program (Fujifilm Medical Systems, USA). The intraclass correlation coefficient between all examiners was 0.931 (*P* < 0.001) for the CS and 0.958 (*P* < 0.001) for the DS.

One week after the calibration process, random digital X-ray images of 53 maxillary molar teeth (159 roots) acquired from the same database were examined by the 4 observers, once by a CS (HP Elite display e243M, a 23.8-inch IPS, MicroEd LED Backlit, 1920 × 1080 @ 60 Hz screen) and again 2 months later (as suggested by previous dental X-ray research, intervals of at least one week and up to several months have been used to overcome memory bias. 10, 13) by a DS (NEC MD213MG, a 21.3”, 3 MP, 2048 × 536 medical image screen). The viewing conditions were similar both times, including a darkened room at approximately the same time of day (morning). The observers were instructed not to adjust the image properties (e.g., the contrast and exposure) in any way. The goals were as follows: A. to identify their ability to observe and diagnose apical periodontal ligament (PDL) widening, which has been defined as at least twice the width of the lateral PDL along the root, by giving the image a score of either “0” (not widened) or “1” (widened); and B. to identify their ability to observe the tooth’s root apices, which they scored “1” (clearly visible) and “0” (not clearly visible).

SPSS version 28.0.1.1 was used for the statistical analysis.

Statistical analysis of the intra- and interobserver comparisons was performed by the kappa test for dichotomic variables and intraclass correlation measures for continuous variables.

For all analyses, the level of significance was set at *P* < 0.05.

This study was approved by the Institutional Review Board of the Israel Defense Force Medical Corps, approval number #2004–2019. Informed consent was waived for this anonymized retrospective analysis.

## Results

**Fig. 1:**
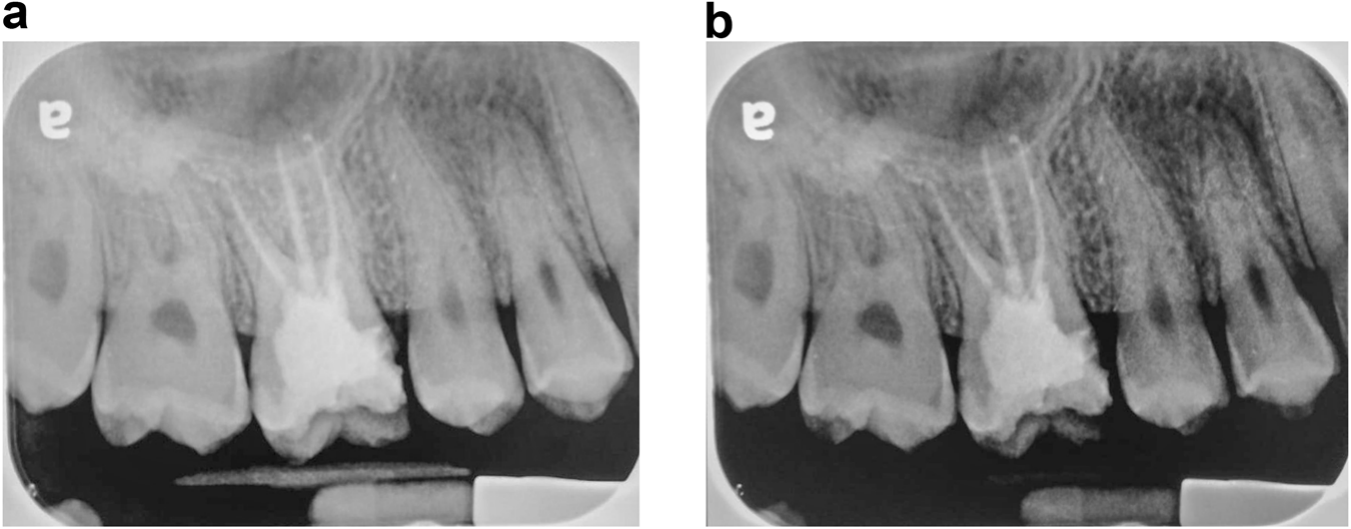
A periapicl x-ray of tooth #16, in both commercial screen and a diagnostic screen, photographed using a digital camera. **a**, **b** Tooth #16: The PDL of the buccal roots was identified as “widened” when using DS and not “not widened” when using CS (**a** = CS, **b** = DS).

PDL widening test: The interobserver agreement (Tables 1, 2) for the PDL widening test for the observers HS and IS was 0.527 (*P* < 0.001) for the CS and 0.662 (*P* < 0.001) for the DS. An observer IS was able to identify 17 patients (17 roots, 17.3%) with widened PDLs via the DS, which were classified as “not widened” in the CS. He classified a total of 38 patients (31.4%) as “widened” with the DS compared to 23 (19.0%) with the CS. The kappa value for the comparison between the screens for the IS PDL test was 0.592 (*P* < 0.001). The observer HA found 12 patients (13%) with a widened PDL when using the DS, which she classified as “not widened” with the CS. She found that 34.8% of the patients had a widened PDL with the DS, while only 26.1% had a widened PDL with the CS. The kappa value for the comparison between the screens for the PDL test was 0.593 (*P* < 0.001). An example of an image in which the PDL was considered widened according to the DS but not by the CS in Tooth #17 is given in Fig. 1.

**Table 1:** Comparison of intraobserver agreement between diagnostic screens and commercial screens (kappa and P values^*^).

A.	For PDL widening				
	Diagnostic Screen				
Commercial Screen	Observer	AL	AS	IS	HA
	AL	0.627			
	AS		0.511		
	IS			0.592	
	HA				0.593

B.	For observing root apices				
	Diagnostic Screen				
Commercial Screen	Observer	AL	AS	IS	HA
	AL	0.435			
	AS		0.241		
	IS			0.736	
	HA				0.400

^*^P < 0.001 for all.

**Table 2:** Interobserver agreement for diagnostic screens and commercial screens (kappa and *P*-values^*^).

A.	For PDL widening			
Observer	AL	AS	IS	HA
AL	-	DS:0.499 CS:0.650	DS: 0.51 CS: 0.612	DS: 0.440 CS: 0.499
AS	-	-	DS: 0.595 CS: 0.470	DS:0.421 CS:0.631
IS	-	-	-	DS: 0.662 CS: 0.527

B.	For observing root apices			
	AL	AS	IS	HA
AL	-	DS: 0.389 CS: 0.551	DS: 0.603 CS: 0.402	DS:0.443 CS: 0.515
AS	-	-	DS: 0.452 CS: 0.373	DS: 0.318 CS: 0.602
IS	-	-	-	DS: 0.595 CS: 0.413

^*^P < 0.001 for all.

**Fig. 2:**
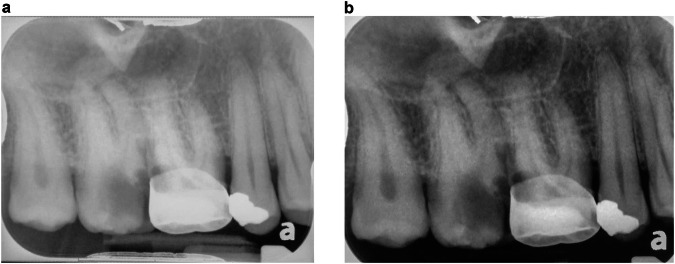
A periapicl x-ray of tooth #16, in both commercial screen and a diagnostic screen, photographed using a digital camera. **a**, **b** Tooth #16: The root apices were not visible when using the CS, while the DS identified their apices (**a** = CS, **b** = DS).

Root apices test: The interobserver agreement (Tables 1, 2) for observers HA and AL was 0.515 (*P* < 0.001) for the CS and 0.443 (*P* < 0.001) for the DS. The observer AL was able to detect the root apices in 123 patients (77.4%) when using the DS and only 89 (56%) when using the CS. The abilities of observer HA also differed depending upon the screening system: she was able to detect the root apices 133 times (83.6%) when using the DS, and these figures decreased to 106 (66.7%) when she used the CS. The overall kappa value for the comparison between the screens was 0.400 (*P* < 0.001) for Observer HA and 0.435 (*P* < 0.001) for Observer AL (Tables 1, 2). An example of an image in which the root apices were not visible using the CS, while the DS identified their apices is given in Fig. 2.

## Discussion

Currently, the best radiological technique for evaluating the periapical area is CBCT. However, the ALARA principles require strict case selection. Thus, referring all patients to a CBCT scan prior to treatment is not recommended. In most clinical situations, the clinician must make a decision based on a combination of clinical and 2D X-ray data. During this research, we attempted to create conditions that mimic the clinical scenario in which the clinician is guided only by an evaluation of the periapical X-ray.

The maxillary posterior region poses several challenges in dental radiography. It is a complex area to interpret on 2-dimensional X-rays due to the superposition of anatomical structures, such as the border of the maxillary sinus, the zygomatic arch, and fused and closely positioned roots [8]. The current analysis compared the ability of DS and CS imaging to detect PDL widening and visualize the root apices of maxillary molar teeth, and the results were significantly in favor of DS for these purposes.

Identification of PDL widening is crucial in endodontic decision-making since it might serve as a diagnostic criterion for the need for endodontic intervention [11]. To observe the PDL in the apical third of the tooth’s root, which is the most important part of the PDL when looking for the presence of widening in endodontics, one must first observe the entire length of the root, even when it is superimposed by other anatomical structures. A 2017 study [1] revealed that medical calibrated screens performed better in the identification of incipient and recurrent caries lesions. A series of studies conducted at the University of Oulu [8] revealed that DS may improve observer performance in the detection of pathology in panoramic radiographs regardless of the room illuminance level. Hirschorn et al. [7] listed 2 major reasons for preferring DS over CS: 1. a commercial grade monitor does not show all of the critical anatomical information, and as the name implies, the CS monitors are intended to display documents to appear like a printed page in office settings; 2. many commercial grade monitors do not have the required dynamic range, while medical grade monitors are calibrated in a way that takes into account the room light and maps the lowest pixel value to the detectable output. A medical-grade monitor typically adjusts the output to compensate for start-up variations in output; it can usually keep a record and keep track of its calibration, and it is typically certified.

The results of the current analysis indicated that there are several important differences in favor of the DS over the CS for visualizing PDL widening and root apices. First, the observers were able to identify more cases of widened PDLs when using the DS (31.4% compared to 17.4% for one observer and 34.8% compared to 21.7% for the other), meaning that cases that were identified by the CS as “not widened” were identified as “widened” by the DS. This could determine an entirely different treatment plan. Second, one observer was able to detect the root apex in 123 patients (77.4%) when using the DS compared to only 89 (56%) when using the CS. Another observer was able to detect the root apices 133 times (83.6%) when using the DS and only 106 (66.7%) when using the CS. Third, the interobserver agreement for the PDL widening test was 0.527 (*P* < 0.001) for the CS and 0.662 (*P* < 0.001) for the DS, indicating that a more suitable screen might improve interobserver agreement (although it did not improve interobserver agreement for the visibility test of apices for all of the observers). The issue of interobserver agreement has long been debated in the endodontic radiography literature. In 1972, Goldman et al. [12] showed that even very experienced observers agreed in only 47% of the cases when they examined 253 random X-rays. In 2011, Tewary et al. [12, 13] repeated Goldman et al.‘s experiment using 150 digital random X-rays. The former authors also reported an average kappa value of 0.5. Omer et al. [14] found limited value of radiographs when studying certain aspects of the root canal system and concluded that poor and average intra- and interobserver values in such studies are to be expected. Similar findings emerged in the current study. Since the interobserver agreement was low for some observers, those for whom the kappa value ranged between moderate and substantial were selected. Overall, however, the interobserver agreement in the current study was consistent with that of past studies. Nevertheless, the finding that the DS was able to improve the kappa value for the PDL widening test might suggest that screens that are better in terms of calibration or more suitable for medical purposes might improve the clinician’s ability to detect and diagnose anatomical structures and pathological conditions. Such screens might serve as a better research tool for endodontic radiography studies and improve past kappa values in endodontic radiography research, as shown in this study.

The main limitation of this research is that there is no “gold standard”, such as a CBCT scan, for comparison. This was done as a simulation of the clinical scenario, as stated before.

Additional studies are warranted to evaluate the influence of screen capability on clinician decisions to refer patients to cone beam computed tomography for cases that are difficult to interpret.

In conclusion, the DS showed a significantly greater ability to identify PDL widening and root apices than did the CS in the posterior maxillary region.

## Data Availability

The datasets used and/or analyzed during the current study are available from the corresponding author on reasonable request.
